# Effects of Nitrogen Addition on the Drought Susceptibility of the *Leymus chinensis* Meadow Ecosystem Vary with Drought Duration

**DOI:** 10.3389/fpls.2018.00254

**Published:** 2018-02-27

**Authors:** Baoku Shi, Yunbo Wang, Bo Meng, Shangzhi Zhong, Wei Sun

**Affiliations:** ^1^Key Laboratory for Vegetation Ecology, Ministry of Education, Institute of Grassland Science, Northeast Normal University, Changchun, China; ^2^Key Laboratory of Grassland Resources, Ministry of Education, College of Grassland, Resources and Environment, Inner Mongolia Agricultural University, Hohhot, China

**Keywords:** climate change, nitrogen deposition, extreme drought, phytomass, ecosystem C exchange, ecosystem functions, saline-alkaline grassland

## Abstract

It is not clear yet how extreme drought and nitrogen (N) deposition influence grassland ecosystem functions when they are considered together, especially in complex field conditions. To explore the response of the *Leymus chinensis* meadow ecosystem to manipulated extreme drought (45 days), N addition and their interaction, we measured leaf photosynthetic characteristics, aboveground phytomass on the community level and ecosystem C exchange in different treatments at the middle and the end of the drought period. The extreme drought treatment decreased the leaf net CO_2_ assimilation rate and ecosystem C exchange [gross ecosystem productivity (GEP), ecosystem respiration and net ecosystem CO_2_ exchange]. In contrast, the N addition treatment increased aboveground phytomass, GEP and net ecosystem CO_2_ exchange. The effects of N addition on the drought susceptibility of the *L. chinensis* meadow ecosystem varied with drought severity. The N addition treatment alleviated drought-induced suppression of CO_2_ exchange at the leaf and ecosystem levels in the middle of the drought period, whereas it exacerbated drought-induced suppression of the CO_2_ exchange and aboveground phytomass on the community level at the end of the drought period. Given that dominance by *L. chinensis* is a characteristic of the studied ecosystem, knowledge of the traits of *L. chinensis* and its response to multiple global change drivers will be crucial for predicting future ecosystem functions. Furthermore, increasing N deposition may affect the response of the *L. chinensis* meadow ecosystem to further droughts by increasing carbon allocation to roots and therefore root–shoot ratios.

## Introduction

Anthropogenic activities strongly influence environmental change, including discrete climate extremes (e.g., drought and torrential rainfall) and chronic environmental changes (e.g., N deposition and increasing CO_2_ concentration) (IPCC, 2013; Zhu et al., 2016). Many terrestrial ecosystems, including grasslands, face unprecedented shifts in these environmental conditions and are affected by discrete climate extremes and chronic environmental changes, as well as their interactions, with unknown consequences for ecosystem functions and services (livestock forage production, biodiversity preservation and C sequestration) (Smith, 2011, Lee et al., 2014; Hufkens et al., 2016; Smith et al., 2016). Thus, understanding how grassland ecosystems respond to multiple global change drivers is important.

In temperate regions, drought events are expected to increase in intensity and/or duration due to climate change (IPCC, 2013). Both linear and non-linear models show a strong correlation between drought and grassland productivity (Knapp et al., 2017). Drought induces decreases in the soil water content and increases in plant water deficit, which cause subsequent decreases in the leaf carbon assimilation rate and soil available nutrients, leading to plant N limitation and earlier senescence and mortality of tissues, further reducing plant aboveground productivity and net ecosystem CO_2_ exchange (NEE) (Durand et al., 2010; Yi et al., 2015; Hofer et al., 2016; Hoover et al., 2017). Grassland ecosystems face both extreme drought events and other global change drivers, of which increasing N deposition strongly influences aboveground productivity and carbon cycles (Niu et al., 2010; Ladwig et al., 2012). Atmospheric N deposition has increased by nearly five times in China from 1901 to 2005 and is expected to further increase in coming decades (Lu et al., 2012). Plants are commonly N-limited in grassland ecosystems (LeBauer and Treseder, 2008). As such, enhanced atmospheric N deposition increases soil available N, alleviates plant N limitation and stimulates leaf photosynthesis (Evans, 1989), further increasing plant aboveground productivity and NEE (Niu et al., 2010). The combination of extreme drought and N addition may not act additively but produce non-additive interrelated effects on ecosystem responses (Meyer-Grünefeldt et al., 2015). Many studies have suggested that N addition could make grassland ecosystems more vulnerable to extreme drought events (Xu et al., 2014; Song and Yu, 2015). This is because N addition causes a disproportionate increase in aboveground phytomass and more evaporative water loss, thus increasing the likelihood of drought stress (Friedrich et al., 2012; Southon et al., 2012). However, the effects of N addition on drought sensitivity of grassland ecosystems may depend on the drought intensity and/or duration (Knapp et al., 2008; Greaver et al., 2016). In the early stages of drought, the amount of water in the soil is relatively sufficient, which may induce mild drought stress on plants. Meanwhile, N addition associated with greater leaf N content and water use efficiency could partially compensate for drought-induced suppression. With the intensification of drought stress, N addition is likely to exacerbate drought effects due to higher phytomass, related greater water consumption and a lower root–shoot ratio associated with a reduced ability to acquire new water sources. However, this hypothesis has not been tested yet.

Furthermore, most studies on the effects of extreme drought and/or N addition focus at a single level of biological organization, e.g., at the physiological level (Flexas et al., 2008) or on the community level (Hoover et al., 2015). These manipulation experiments focusing on a single level cannot give a complete picture of the effects of multiple stressors on grassland ecosystems (Reyer et al., 2013). The effects of drought and/or N addition are predicted to begin at the gene expression or metabolomics level and translate up to higher hierarchical levels (Smith et al., 2009; Peñuelas et al., 2017). Here, we focus on how leaf photosynthetic characteristics respond to drought and/or N addition and whether these individual level physiological responses can translate to community and ecosystem level responses.

*Leymus chinensis* meadow steppe, located on the eastern edge of the Euro-Asian grassland, is the most typical grassland type in northeast China (Zhu, 2004; Zhang et al., 2013). Soil water content and soil N availability are the primary limiting factors for plant growth in *L. chinensis* meadow steppe because of the limited rainfall and widespread distribution of saline alkali soil (Wang et al., 2015). The extreme drought and N addition treatments were performed in a *L. chinensis* meadow steppe, which provided an opportunity to explore the response of leaf photosynthetic characteristics and ecosystem functions (species diversity, aboveground productivity and ecosystem C exchange) to these stressors under *in situ* conditions. We hypothesized that (1) leaf carbon assimilation, aboveground phytomass and ecosystem C exchange decline in response to extreme drought; (2) leaf carbon assimilation, aboveground phytomass and ecosystem C exchange increase under the N addition treatment; and (3) N addition alters the drought sensitivity of the *L. chinensis* meadow ecosystem, and the effects are likely to vary with drought duration.

## Materials and Methods

### Ethics Statement

No specific permissions were required for the field studies described, because the Songnen Grassland Ecological Research Station is a department of the Northeast Normal University. No specific permissions were required for the study either, as it was conducted in accordance with the guidelines set by the Northeast Normal University. No specific permissions were required for the locations or the activities. No location was privately owned or protected in any way, and the field studies did not involve endangered or protected species.

### Study Area

The study was conducted in the Songnen Grassland Ecology Research Station (44°45′N, 123°45′E), Jilin Province, northeastern China. The study site has a temperate semiarid monsoon climate. The mean annual temperature is 6.4°C (1950–2004). The mean annual precipitation is 471 mm (1950–2004), which is concentrated in the summer (June–August). The duration of the frost-free season is 150 days. The studied grassland has a sodic saline meadow soil with a pH value of 8.0–9.0, soil organic carbon content of 2.0% and soil total nitrogen content of 0.15% (Wang et al., 2015). The vegetation of the studied meadow steppe is primarily composed of *L. chinensis*; *Phragmites australis*, *Chloris virgata*, and *Kalimeris integrifolia* are also present at lower densities (Zhang et al., 2013; Cui et al., 2015).

### Experimental Design

Before 2009, the studied grassland was lightly grazed by large livestock herds dominated by cattle and sheep, and was mowed each fall. In 2009, the studied grassland, with an area of 100 m × 100 m, was fenced to avoid grazing and mowing. Within the fenced grassland, six experimental blocks were randomly established in April 2011. Each block consisted of two 10 m × 10 m plots, which were separated by a 5 m wide buffer strip. Each plot was randomly assigned to the fertilized or unfertilized treatments. The atmospheric N deposition in the temperate grassland ecosystems in northern China was as high as 2.7 g N m^-2^ yr^-1^ in recent 10 years (Bai et al., 2010). The studied grassland is surrounded by cropland which is often over-fertilized by N; thus, there are probably large emissions of nitrogenous pollutants by volatilization, denitrification, etc., and atmospheric N (re)deposition is more significant than in other grassland areas (He et al., 2007). Moreover, the community saturation N deposition rate was approximately 10.5 g N m^-2^ yr^-1^ in this temperate grassland ecosystem (Bai et al., 2010). As such, N, in the form of urea, was applied five times yearly (May–September) at a rate of 10 g N m^-2^ yr^-1^ to the N addition treatment plots from 2011 to 2016.

*Leymus chinensis*, the dominant species of the Songnen meadow steppe, is a rhizomatous grass that uses the C_3_ photosynthetic pathway (Zhong et al., 2017). To avoid summer high temperatures, it has a relatively short growing season and completes its breeding season before July. The daily growth rate of *L. chinensis* peaks in the middle of June, then begins to decline, and it stops growing in late July (Supplementary Table S1; Zhu, 2004). An extreme drought event was simulated from day 152 (1 June) to day 196 (15 July) in 2015 because the growth of *L. chinensis* in this period is sensitive to the water supply. Two 3 m × 3 m subplots were established within each plot (10 m × 10 m) in 2015 and were randomly assigned to the extreme drought treatment or the ambient precipitation treatment. On the outside edges of the extreme drought subplots, we dug a trench to a depth of 50 cm. To prevent root growing out from the trenched subplots, surface runoff and the lateral flow of soil water, we lined the trenched subplots with a 2-mm corrosion resistant plate and then refilled the soil back into the trench. The extreme drought was achieved using 3.5 m × 3.5 m wide rainout shelters with slanted roofs (1 m tall) composed of transparent acrylic sheets (>90% light permeability). After the simulated extreme drought event, we carried out irrigation (four times) from late July to late August according to the amount of precipitation during the drought period to equalize the annual precipitation. The irrigation was carried out 1 week after the end of the drought treatment. In this week, there was no natural precipitation event; thus, the drought treatment lasted a week longer (to day 202). Our experimental design was fully factorial with four treatments: a N addition treatment (N), a drought treatment (D), a combination of N addition and drought treatment (DN), and a control (C). Soil properties for the four treatments in 2014 are summarized in Supplementary Table S2.

### Micro-climate

Precipitation and air temperature (at a height of 1 m above the ground) were measured hourly by a rainfall recorder and temperature sensor (RG2-M, Onset, Bourne, MA, United States) during the drought period in 2015. The soil temperature and soil water content at a depth of 10 cm in each subplot (N, D, DN and C) in the selected representative block were measured hourly by a soil moisture probe (S-SMC-M005, Onset, Bourne, MA, United States) and a soil temperature probe (S-TMB-M006, Onset, Bourne, MA, United States), respectively, during the growing season (early June to late August) in 2015.

### Leaf Photosynthetic Characteristics

The CO_2_ and water flux at the leaf level and ecosystem level were measured using portable CO_2_ infrared gas analyzers (LI-6400, LI-COR Inc., Lincoln, NE, United States). To reduce the time required for each measurement campaign, two LI-6400 analyzers (IRGA) were used for the gas exchange measurements (one for the leaf level and the other one for the ecosystem level). The net CO_2_ assimilation rate (*A*), stomatal conductance (*g_s_*) and transpiration rate (*T_r_*) of *L. chinensis* were measured on day 173 (day 22 of the drought period) and 196 (day 45 of the drought period) in 2015. For each treatment, 18 individuals (three individuals in each subplot, six replications) were measured for each sampling campaign. For each individual plant, an upper most fully expanded leaf was used for the leaf gas exchange measurements. Measurements were performed from 7:30 to 11:30 AM. To avoid bias from the time of day, we measured six blocks (each block contained four treatments) sequentially. As such, the differences of time of measuring among treatments within a block were negligible. To limit the effects of fluctuations in light intensity and the atmospheric CO_2_ concentration on leaf photosynthetic characteristics, photosynthetically active radiation and the CO_2_ concentration were held at 1500 μmol m^-2^ s^-1^ and 400 μmol mol^-1^, respectively. The temperature and relative humidity in the leaf chamber were maintained at ambient values.

### Ecosystem C and Water Flux

A square aluminum frame (0.5 m × 0.5 m) was inserted into the soil (to a depth of 3 cm) in each subplot in May 2015. Ecosystem C and water fluxes were measured on day 173 and 196 in 2015 using an IRGA with a transparent chamber (0.5 m × 0.5 m × 1.0 m). The chamber was placed on the frame surface during the measurement. For measuring NEE and evapotranspiration (ET), the chamber was set on the frame for 2 min, and the fluxes were determined from changes in the CO_2_ and water vapor concentrations recorded at 10 s intervals. Then, the chamber was vented and placed on the same frame used for ecosystem respiration (ER) measurements. The chamber was covered by an opaque cloth to eliminate light. The ER was determined from changes in the CO_2_ concentration recorded at 10 s intervals for 2 min. Gross ecosystem productivity (GEP) was calculated as ER – NEE. The water-use efficiency (WUE) was calculated as -NEE/ET. In May 2015, one polyvinyl chloride collar (10.4 cm diameter × 6 cm height) was inserted into the soil in each subplot for soil respiration (SR) measurements. The aboveground parts of all plants inside the soil collars were clipped 1 day before the SR measurements. SR was also measured on days 173 and 196 in 2015 using an IRGA. The measurements of NEE, ER, ET, and SR were carried out from 7:30 to 11:30 AM. Ecosystem CO_2_ exchanges were measured sequentially for the six blocks.

### Plant Composition and Phytomass

The vegetation was surveyed on day 196 in 2015 and in August 2016. Three 0.5 m × 0.5 m quadrats in each subplot were randomly selected to survey plant species and an individual number of each species. Using the vegetation survey data, we calculated the species richness and the Shannon-Wiener index (Krebs, 1985):

$$H=-\overset{S}{\underset{i=1}{\Sigma }}P_{i}InP_{i}$$

where *H* is the Shannon-Wiener index, *P*_i_ is the proportion of the individual number for *i* species over the individual number of all species, and *S* is the number of species.

Following the vegetation survey, the aboveground phytomass (both living and dead) was harvested in one of three 0.5 m × 0.5 m quadrats. Because the leaves were not withered completely during the drought period, the aboveground phytomass was not separated into pure biomass and necromass. Two soil cores (5.5 cm diameter × 30 cm depth) were taken to estimate root phytomass using a root corer in the same quadrat. Next, the roots in the soil cores were collected, dried at 60°C to a constant mass, and weighed. The root–shoot ratio was calculated as root phytomass/shoot phytomass.

### Assessment of Drought Impact

The effects of extreme drought on leaf photosynthetic characteristics, phytomass on the community level and ecosystem C exchange in the unfertilized or fertilized plots were quantified as the percentage change in these variables (% Impact-D). Impact-D was calculated from Equation (2):

Impact−D=(XD−XCXC)×100,OR=(XDN−XNXN)×100

where *X_C_*, *X_D_*, *X_N_*, and *X_DN_* are *A*, *g_s_*, *T_r_*, aboveground phytomass, root phytomass, root–shoot ratio, GEP, ER, NEE, ET, WUE, or SR in the C, D, N, and DN plots, respectively.

### Data Analysis

Data were tested for normality using a Kolmogorov–Smirnov test and for variance homogeneity using Levene’s test. The effects of N addition, drought and measuring time on *A*, *g_s_*, *T_r_*, GEP, ER, NEE, ET, and WUE were analyzed using a repeated-measures analysis of variance (ANOVA), with N addition and drought as the between-subjects factors and measuring time as the within-subject factor. Comparisons of the means of response variables related to treatments (C, D, N, and DN) in each measurement campaign were analyzed with an ANOVA combined with Tukey’s *post hoc* test. A paired sample *t*-test was used to assess differences in the change in leaf, community and ecosystem response variables induced by drought (Impact-D) between the fertilized and unfertilized plots. A linear regression analysis was used to examine dependence of the *A*, *g_s_*, and *T_r_* on the soil water content as well as relationships between the GEP and NEE and aboveground phytomass. All statistical analyses were conducted with the SPSS 18.0 software (SPSS Inc., Chicago, IL, United States). Graphs were generated using the SigmaPlot 12.5 software (Systat Software Inc., San Jose, CA, United States).

## Results

### Precipitation and Soil Water Content

**FIGURE 1:**
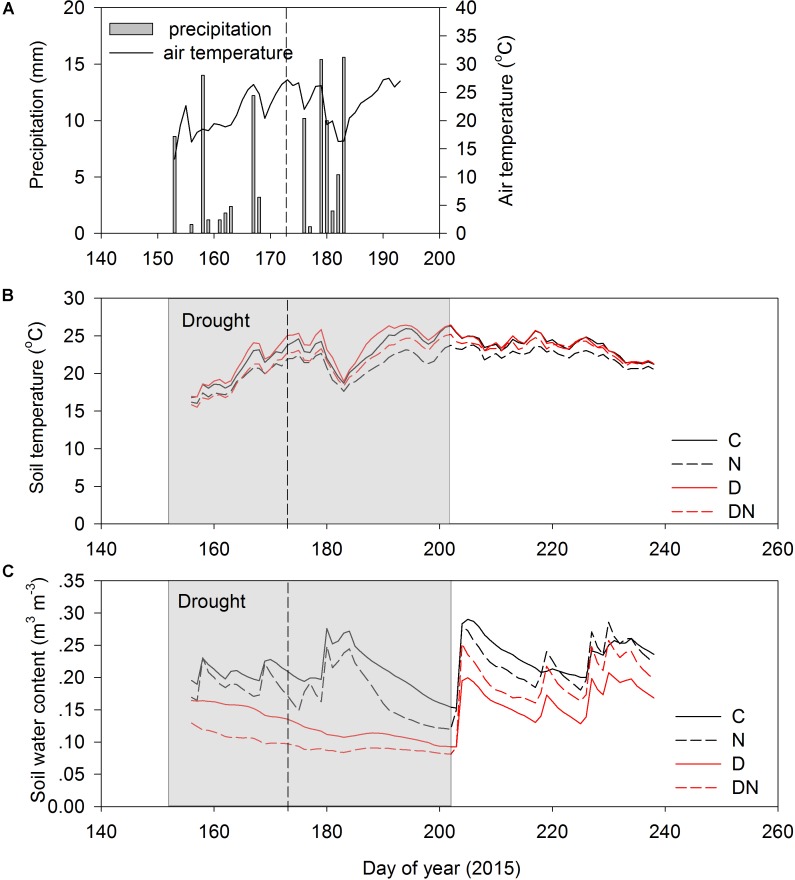
Precipitation **(A)**, soil temperature (0–10 cm depth) **(B)**, and soil water content (0–10 depth) **(C)** for four treatments (C, control; N, nitrogen addition; D, drought; DN, nitrogen addition plus drought) during the growing season in 2015. Shaded area indicates a 51 days extreme drought event (DOY 152 to DOY 202). Vertical dashed lines indicate the middle of the drought period (DOY 173).

The amount of precipitation was 104.6 mm during the extreme experimental drought period (days 152–196; **Figure 1A**). The air temperature varied substantially during the experimental period (**Figure 1A**). As a result of shading effects of greater aboveground phytomass and cover, the soil temperatures (10 cm) in the N and DN plots were lower than in the C and D plots (**Figure 1B**). The seasonal variation of the soil water content in the C and N treatments depended on the occurrence of rainfall events (**Figure 1C**). The N addition, drought and combination of N addition and drought treatment had a strong effect on the soil water content, and the soil water contents in the N, D, and DN plots were 28, 43, and 52% lower than that in the C plots on day 196 (the end of the drought period), respectively.

### Leaf Gas Exchange

**FIGURE 2:**
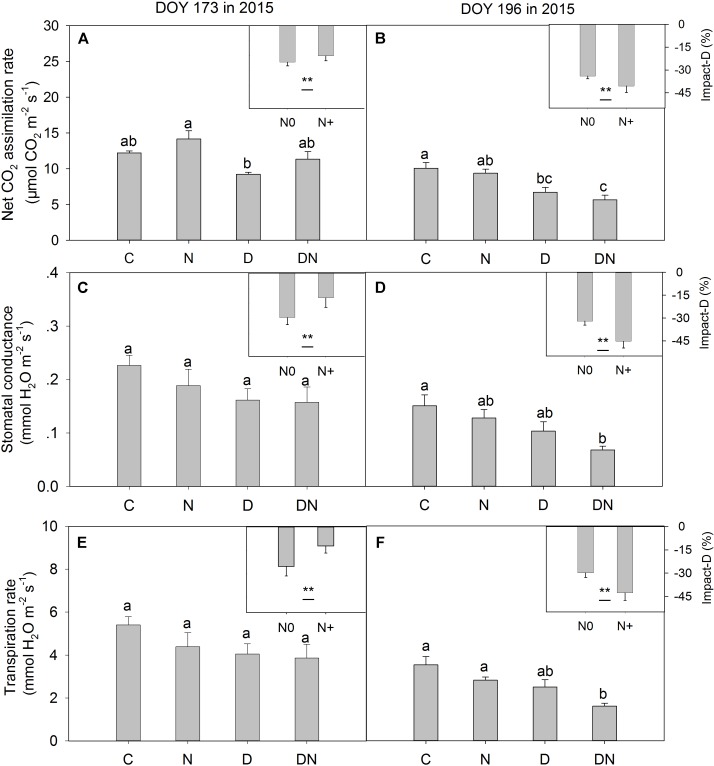
Responses of leaf gas exchange parameters [**(A,B)** net CO_2_ assimilation rate, **(C,D)** stomatal conductance and **(E,F)** transpiration rate] in *Leymus chinensis* to four treatments (C, control; N, nitrogen addition; D, drought; DN, nitrogen addition plus drought) at the middle (DOY 173) and the end (DOY 196) of the drought period. Impact-D = (*X*_D_ – *X*_C_)/*X*_C_ × 100, or = (*X*_DN_ – *X*_N_)/*X*_N_ × 100, where *X* indicates net CO_2_ assimilation rate, stomatal conductance, or transpiration rate. Different letters and asterisk indicate significant differences between the treatments (*P* < 0.05). The error bars represent standard errors of means (*n* = 6).

**FIGURE 3:**
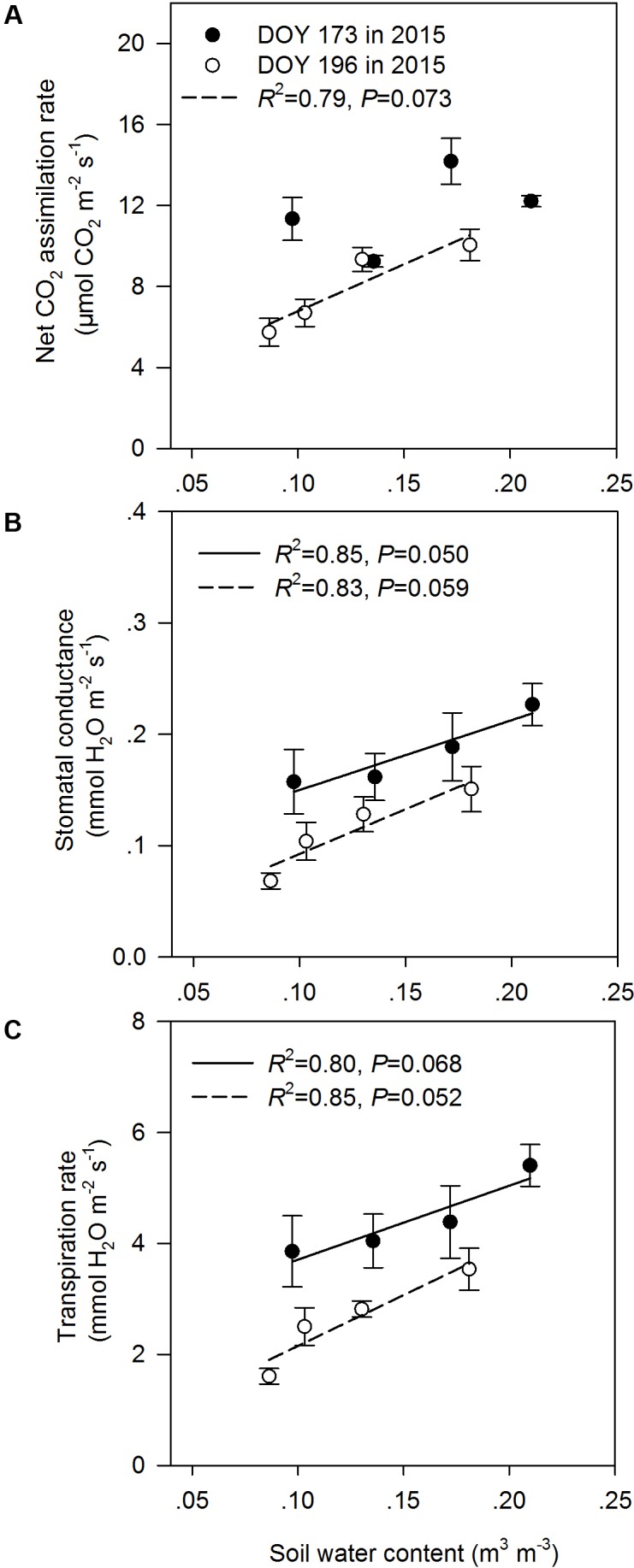
Relationships between leaf gas exchange parameters [**(A)** net CO_2_ assimilation rate, **(B)** stomatal conductance, and **(C)** transpiration rate] and soil water content at the middle (DOY 173) and the end (DOY 196) of the drought period. The error bars represent standard errors of means (*n* = 6).

In the middle of the drought period (day 173, 2015), the N addition, drought and combination of N addition and drought treatments had no significant effects on *A*, *g_s_*, and *T_r_* compared to the C treatment (*P* > 0.05; **Figures 2A,C,E**). At the end of the drought period (day 196, 2015), the drought treatment significantly reduced *A*, regardless of whether it was in the fertilized or unfertilized plots (**Figure 2B**). The DN treatment had the lowest *A*, *g_s_*, and *T_r_* values on day 196 in 2015. The *A*, *g_s_*, and *T_r_* varied with sampling dates, and *A*, *g_s_*, and *T_r_* were lower on day 196 than on day 173 in 2015 (**Figure 2** and Supplementary Table S3). Drought-induced reductions in *A*, *g_s_*, and *T_r_* were lower in the fertilized plots (Impact-D: -20, -16, and -12%) than in the unfertilized plots (Impact-D: -24, -29, and -25%) on day 173 in 2015 (**Figures 2A,C,E**). However, this pattern was different on day 196 in 2015, with greater drought-induced changes in *A*, *g_s_*, and *T_r_* in the fertilized plots (Impact-D: -39, -45, and -43%) than in the unfertilized plots (Impact-D: -33, -32, and -30%) (**Figures 2B,D,F**). On day 173 in 2015, we detected a marginally significant positive dependence of *g_s_* and *T_r_* on soil water content (*P* < 0.1; **Figure 3**), while the relationships between soil water content and *A* were not statistically significant (*P* = 0.531; **Figure 3**). At the end of the drought period, we found a marginally significant positive dependence of *A*, *g_s_*, and *T_r_* on the soil water content (*P* < 0.1; **Figure 3**).

Relationships between GEP **(A)** and ecosystem CO_2_ exchange (NEE) **(B)** and aboveground phytomass across all the treatments except DN treatment (solid line) and all the treatments (dash line) at the end of the drought period (DOY 196 in 2015).

### Ecosystem C Exchange

**FIGURE 4:**
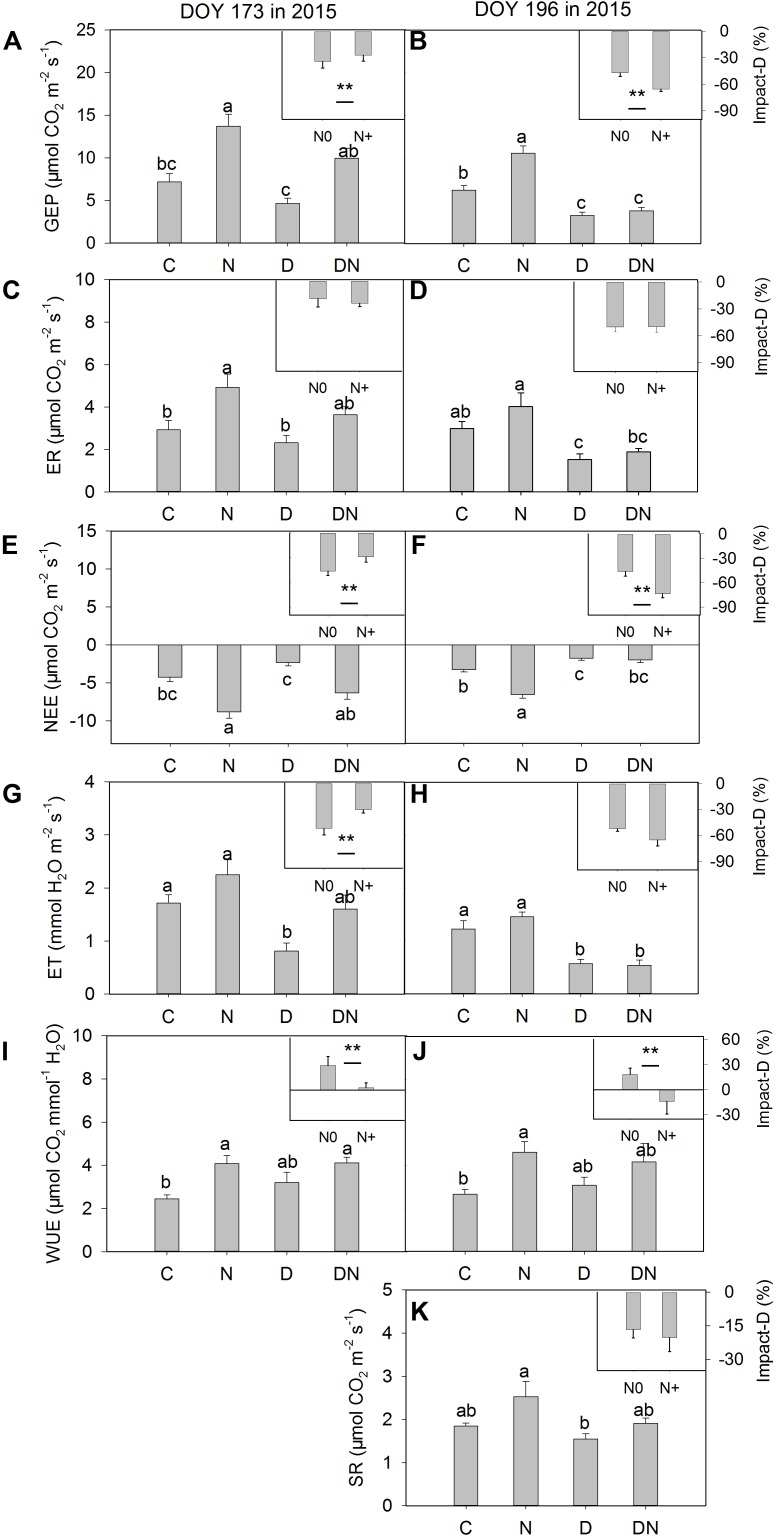
Responses of **(A,B)** gross ecosystem productivity (GEP), **(C,D)** ecosystem respiration (ER), **(E,F)** net ecosystem CO_2_ exchange (NEE), **(G,H)** evapotranspiration (ET), **(I,J)** water-use efficiency (WUE), **(K)** soil respiration (SR) to four treatments (C, control; N, nitrogen addition; D, drought; DN, nitrogen addition plus drought) at the middle (DOY 173) and the end (DOY 196) of the drought period. Impact-D = (*X*_D_ – *X*_C_)/*X*_C_ × 100, or = (*X*_DN_ – *X*_N_)/*X*_N_ × 100, where *X* indicates GEP, ER, NEE, ET, WUE, and SR. Different letters and asterisk indicate significant differences between the treatments (*P* < 0.05). The error bars represent standard errors of means (*n* = 6).

**FIGURE 5:**
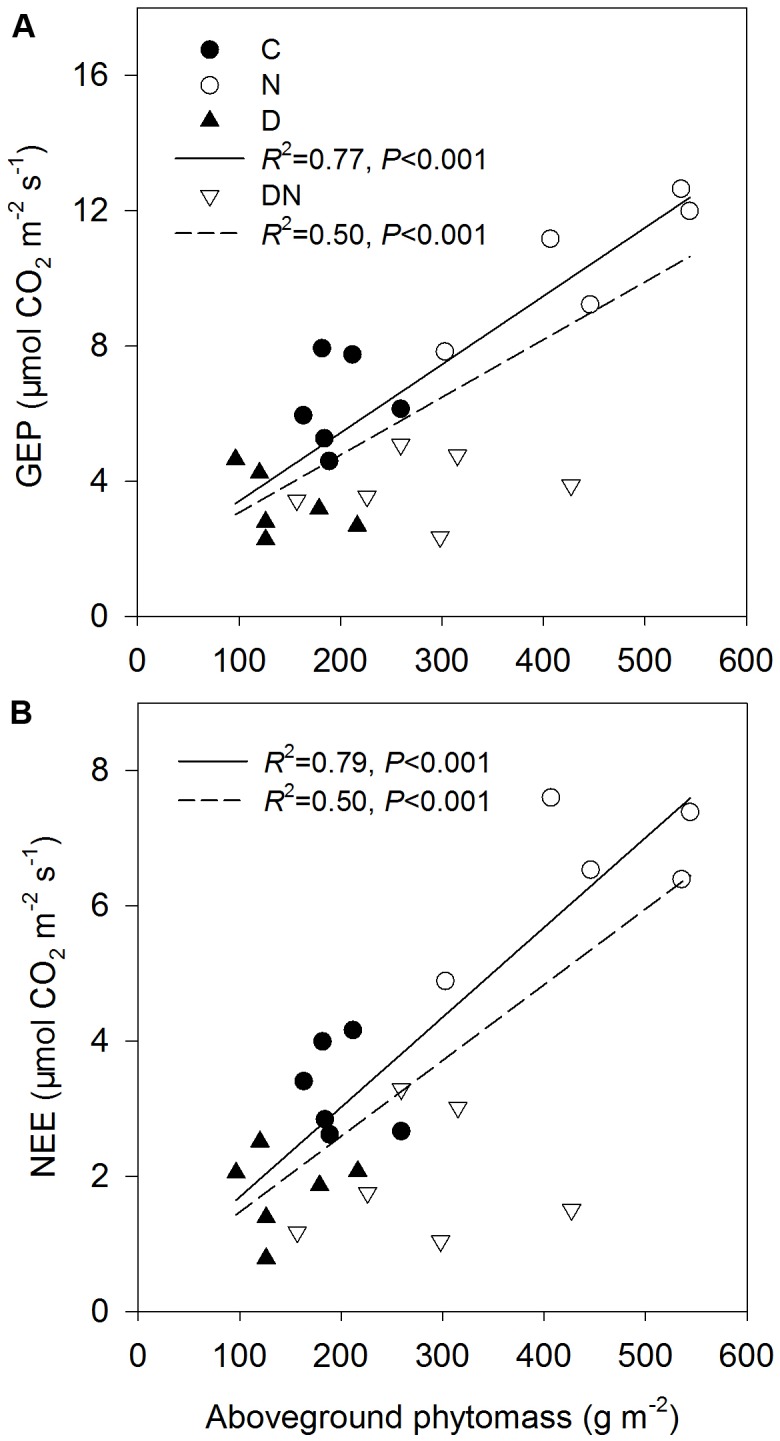
Relationships between gross ecosystem productivity (GEP) **(A)** and ecosystem CO_2_ exchange (NEE) **(B)** and aboveground phytomass across all the treatments except DN treatment (solid line) and all the treatments (dash line) at the end of the drought period (DOY 196 in 2015).

The N addition treatment increased GEP, NEE, and WUE on days 173 and 196 in 2015 (**Figure 4**). The drought treatment had no significant effect on GEP, ER, and NEE on day 173 in 2015, whereas it significantly reduced GEP, ER, and NEE on day 196 in 2015 both in the fertilized or unfertilized plots (**Figure 4**). GEP, ER, NEE, and ET were lower on day 196 than on day 173 in 2015 (**Figure 4** and Supplementary Table S3). The drought-induced reductions in GEP and NEE were lower in the fertilized plots (Impact-D: -27%, -29%) than in the unfertilized plots (Impact-D: -34%, -46%) on day 173 in 2015 (**Figures 4A,E**). In contrast, the drought-induced changes in GEP and NEE in the fertilized plots (Impact-D: -65%, -74%) were greater than in the unfertilized plots (Impact-D: -47%, -46%) on day 196 in 2015 (**Figures 4B,F**). GEP and NEE were positively dependent on the aboveground phytomass (**Figure 5**). The correlations had greater values of *R*^2^ when the DN treatment was excluded from the regression analysis (**Figure 5**).

### Species Richness and Diversity

The number of plant species ranged from a minimum of 4 to a maximum of 8 depending on the treatments and observation date (Supplementary Figures S1A,B). There were no significant treatment differences in 2015 (Supplementary Figure S1A) and 2016 (Supplementary Figure S1B). Despite values of the Shannon-Wiener diversity index that were lower in the N plots compared to the C plots in 2015 and 2016 (Supplementary Figures S1C,D), the differences were not statistically significant. The drought treatment, both on its own and in combination with the N addition treatment, did not significantly affect the diversity index during the observation period.

### Phytomass

**FIGURE 6:**
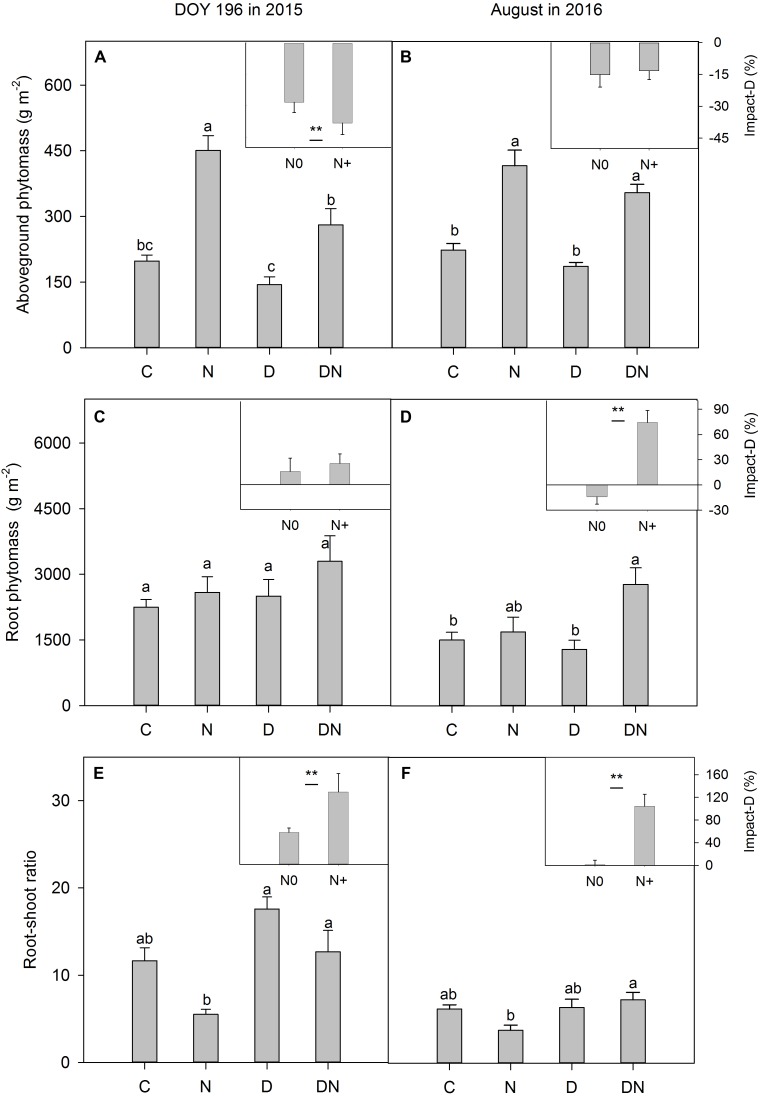
Responses of **(A,B)** aboveground phytomass, **(C,D)** root phytomass and **(E,F)** root-shoot ratio to four treatments (C, control; N, nitrogen addition; D, drought; DN, nitrogen addition plus drought) at the end of the drought period (DOY 196 in 2015) and 1 year after the drought event (August in 2016). Impact-D = (*X*_D_ – *X*_C_)/*X*_C_ × 100, or = (*X*_DN_ – *X*_N_)/*X*_N_ × 100, where *X* indicates aboveground phytomass, root phytomass or root-shoot ratio. Different letters and asterisk indicate significant differences between the treatments (*P* < 0.05). The error bars represent standard errors of means (*n* = 6).

Compared to the C plots, aboveground phytomass in the N plots increased by 128% on day 196 in 2015 (**Figure 6A**) and 86% in August 2016 (**Figure 6B**). Despite aboveground phytomass values that were lower (compared to the C plots) in the D plots, the differences were not significant in both 2015 (**Figure 6A**) and 2016 (**Figure 6B**). For fertilized plots, the drought treatment significantly reduced aboveground phytomass on day 196 in 2015 (**Figure 6A**). There were no significant differences in aboveground phytomass between the N and DN plots in 2016 (**Figure 6B**). Differences in the drought-induced reduction in aboveground phytomass between the fertilized and unfertilized plots were significant on day 196 in 2015, but not in August 2016 (**Figures 6A,B**). The drought treatment induced an increase in root phytomass, but the differences were not significant between the C and D plots or N and DN plots on day 196 in 2015 (**Figure 6C**). However, the percentage of the drought-induced increase in root phytomass was greater in fertilized plots than in unfertilized plots in August 2016 (**Figures 6C,D**). On day 196 in 2015, as a result of drought impacts, the root–shoot ratio in the unfertilized and fertilized plots increased by 57 and 129%, respectively (**Figure 6E**). Compared to the N addition treatment, drought caused the root–shoot ratio in the fertilized plots to increase by 104% in August 2016 (**Figure 6F**).

## Discussion

### Leaf Photosynthetic Characteristics

The drought treatment decreased *A* on day 196 in 2015 (the end of the drought period), which was mainly attributed to the fact that the shortage of soil water induced a limitation of CO_2_ through the closure of stomata and metabolic constraints (**Figure 2**; Flexas et al., 2008). The above reasoning was also supported by the results inferred from our linear regression analysis (**Figure 3**). The regression model suggested that *A* was controlled by the soil water content across different treatments. The drought-associated severe soil water shortage had negative effects on the N metabolism by regulating the activities of key enzymes involved in N assimilation and catabolism and led to damage to cell membranes and a reduction of photosynthetic capacity (Xu and Zhou, 2006). Moreover, the studied grassland has a sodic saline meadow soil with relatively high electrical conductivity (Supplementary Table S2) and degree of salinity, which may exacerbate the drought effect due to reduced soil available water. The soil water potential should be measured during the drought period in further research to understand the response mechanism of leaf photosynthetic characteristics to extreme drought. In general, N addition stimulates the net carbon assimilation rate as a result of the increase in leaf chlorophyll content (Evans, 1989). However, leaf carbon assimilation was not affected by N addition during the observation period in this study. Leaf carbon assimilation is sensitive to variations in light intensity, atmospheric moisture, and temperature (Creese et al., 2014). As such, the stimulating effect of the N addition treatment on *A* might be not captured from the “snapshot” leaf gas exchange measurements.

The N addition treatment alleviated drought-induced suppression of *A*, *g_s_*, and *T_r_* in the middle of the drought period, whereas it exacerbated drought-induced suppression of these photosynthetic characteristics at the end of the drought period. This is closely related to the change in soil water content during the drought period, seasonal changes in air temperature and the growth strategy of *L. chinensis*. In the early stages of drought, the amount of water in the soil was relatively sufficient. N addition could not only stimulate the net carbon assimilation rate (Evans, 1989) but also increase the drought tolerance of plants by preventing cell membrane damage and enhancing osmoregulation (Saneoka et al., 2004). Consequently, when the N addition and drought were combined, there was a reduced drought impact on leaf photosynthetic characteristics in this period. From the middle to the end of the drought period, the drought treatment did not cause a sharp decrease in soil water content, especially in the fertilized plots, which indicated the soil water contents were nearing the wilting point for *L. chinensis*. Compared to the D treatment, the plants in the DN treatment reached wilting point earlier and suffered a longer period of drought stress because of greater aboveground biomass associated higher transpiration. Meanwhile, the higher temperatures and radiation in July may have exacerbated drought stress in the DN communities that were already under pressure toward the end of the drought period. Furthermore, the soil water shortage is generally related to a reduction in plant-available soil N (Durand et al., 2010; Hofer et al., 2016). Thus, the N addition exacerbated drought-induced suppression of leaf photosynthetic characteristics in the second half of the drought event.

### Ecosystem C and Water Flux

N addition stimulated plant growth and consequently enhanced GEP and net C uptake in this study. This is consistent with the findings of many others (Niu et al., 2010; Yan et al., 2011; Estop-Aragonés et al., 2016). Over the 4 years, N addition significantly stimulated growing-season net C uptake, on average, by 27% in a typical temperate steppe (Niu et al., 2010). Estop-Aragonés et al. (2016) also found that the manipulated N deposition treatment increased CO_2_ fluxes and GEP in mesocosms of 14 European peatlands. In contrast, drought decreased GEP, ER and net C uptake, which is in line with a synthesis study suggesting that experimentally reduced precipitation suppressed aboveground net primary production and net C uptake (Wu et al., 2011). The response of GEP and net C uptake to different manipulated precipitation and N addition treatments depends on the changes in aboveground phytomass and species composition (Wu et al., 2011; Yan et al., 2011; Wang et al., 2015). However, the studied grassland was dominated by *L. chinensis*, which caused a non-significant change in functional groups under different treatments. Thus, the physiological traits of dominant species may play an important role in ecosystem C exchange (Wang et al., 2007). This inference was supported by the result of our linear regression analysis. The values of *R*^2^ of the linear model for explaining the variation of GEP and net C uptake across all the treatments except the DN treatment were higher compared to those across all the treatments (**Figure 5**). It is noticeable that the aboveground phytomass in the DN treatment was still higher than that of the C or D treatment, although drought-induced suppression was exacerbated by the N addition treatment. The reason could be that compared to aboveground phytomass, *A* was more sensitive to the decrease in the soil water content and the response of aboveground phytomass on the decrease in the soil water content may have a time delay. As such, the relatively low GEP and net C uptake in the DN treatment may be mainly attributed to the decrease in *A*. Although the response of ecosystem C exchange to N addition, drought and the combination of N addition and drought treatments was in line with expectations, caution is required for the interpretation of these results as these were only snapshots of the drought response processes.

Consistent with the previous study (Tian et al., 2016), we found that the N addition treatment increased WUE during the measurement period. However, the combination of N addition and drought treatment had no significant effects on WUE compared to the D treatment during the observation period (*P* = 0.27, 0.55; **Figures 4I,J**), which was contrary to our expectation. As such, the fact that N addition treatment alleviated drought-induced suppression of the CO_2_ exchange at leaf and ecosystem levels in the middle of the drought period cannot be attributed to the change in WUE.

### Phytomass Production and Allocation

N addition often has a fertilization effect, which increases aboveground phytomass and increases light limitation (reduced transmission of photosynthetically active radiation to ground level), further causing declines in plant diversity (Borer et al., 2014; Hautier et al., 2014). Consistent with the results of previous studies (Henry et al., 2015; Moinet et al., 2017), we found that the N addition treatment stimulated aboveground phytomass production. However, the diversity index was not affected by the treatments of drought, N addition and their interaction. It is probable that *L. chinensis* has strong adaptability and tolerance to drought, waterlogging and salt-alkaline stress and is the absolute dominant species in our study area (Chen and Wang, 2009; Wang D. et al., 2017). Therefore, the species richness and Shannon-Wiener index were very low in all treatments. The drought-induced reduction in aboveground phytomass has been extensively reported (VanderWeide and Hartnett, 2015; Knapp et al., 2017). Our results showed that the D treatment decreased the aboveground phytomass, whereas this effect was not statistically significant, which was contrary to our expectation and inconsistent with the results of previous studies. This indicates that the aboveground productivity of *L. chinensis* meadow was highly resistant to a single extreme drought event.

Drought-induced suppression of aboveground phytomass was exacerbated by the N addition treatment. Our result was consistent with previous studies (Friedrich et al., 2012; Southon et al., 2012; Dziedek et al., 2016). The accelerated aboveground productivity of a perennial grass (*Molinia caerulea*) under N fertilization resulted in higher drought susceptibility (Friedrich et al., 2012). In a lowland heath, N appeared to affect the sensitivity of *Calluna vulgaris* to climate stress, with greater levels of shoot browning in plots receiving N addition during dry years (Southon et al., 2012). The shortage of soil water induced a reduction in the growth rate because the meristems become less active and because of lower *A*, decreases in leaf expansion, and a shift in C allocation (**Figure 6**; De Boeck et al., 2016), which eventually caused earlier leaf senescence, mortality of tissues and reduction in aboveground phytomass production. Because the leaves were not withered completely during the drought period, and pure biomass and necromass were difficult to separate, we could not assess differences in drought-induced tissue senescence between the fertilized and unfertilized treatments. The effects of drought on plant aboveground productivity in the fertilized or unfertilized plots could be underestimated in this study.

In August 2016 (1 year after the drought event), the effects of drought on aboveground phytomass were negligible both in the fertilized or unfertilized plots. This may be attributed to plants compensatory growth following release from stress (Retuerto and Woodward, 2001). On the other hand, as a result of drought impacts, the root phytomass in the unfertilized and fertilized plots increased by 11 and 47%, respectively, which may contribute to the post-drought recovery of grassland productivity. The fresh and old photosynthetic products in aboveground phytomass can be allocated to roots to obtain more available water under extreme drought stress (**Figure 6**; Hasibeder et al., 2015). More aboveground phytomass in the fertilized plots could stimulate this effect. An increase in the root–shoot ratio and root phytomass in the DN treatment at the end of the drought period in 2015 may facilitate the enhancement of clonal expansion (Zhu, 2004) and increase the water obtained after rewetting, which promoted recovery in aboveground phytomass. The results of a study in the Mongolian steppe have also suggested that increasing N deposition enhanced post-drought recovery of grassland productivity, and the increase in productivity was associated with an increase in shoot emergence of a perennial herb, *Artemisia adamsii* (Kinugasa et al., 2012). It is noticeable that this effect (drought-induced increase in root phytomass and the root–shoot ratio in the fertilized plots) persisted 1 year after the drought treatment. This response has not been found in other studies, as available data on the effects of the combination of extreme drought and N addition on grassland ecosystems are limited. Nevertheless, grasses experiencing drought can retain a long-lasting stress imprint (Walter et al., 2011; Fuchslueger et al., 2016). Some studies reported that the projected increase in the frequency of drought and rewetting events could enhance the resistance of organisms to similar disturbances due to improved photoprotection (Walter et al., 2011, 2013). Wang S. et al. (2017) also found that exposure to inundation or drought conditions can induce physiological or morphological changes in plants that improve tolerance for either extreme condition later. Our results show that N addition exacerbated drought-induced suppression of aboveground phytomass under extreme drought stress, but may enhance drought resistance to further drought events due to increased root phytomass and an increased root–shoot ratio. More research is needed to confirm this inference.

Similar to leaf gas exchange and GEP and net C uptake, the effect of drought on aboveground phytomass was exacerbated by the N addition treatment at the end of the drought period. These results suggest that N addition enhances the drought susceptibility of the *L. chinensis* meadow ecosystem at different hierarchical levels under extreme drought stress, including leaf-level responses (*A*, *g_s_*, and *T_r_*), community-level responses (aboveground phytomass) and ecosystem-level responses (GEP and NEE). The response of ecosystem functions at higher ecological levels may be resulted from that of plant ecophysiological responses at the lower hierarchical levels (Hoover et al., 2014; Hoover et al., 2017).

## Conclusion

The N addition treatment alleviated drought-induced suppression of the CO_2_ exchange at leaf and ecosystem levels in the middle of the drought period, whereas it exacerbated drought-induced suppression of the CO_2_ exchange and aboveground phytomass on the community level with prolonged drought. The traits of *L. chinensis* and its ecophysiological response at the lower hierarchical levels to multiple global change drivers determined the response of ecosystem function at higher ecological levels. These findings enable us to better understand the effect of the combination of different global change drivers on *L. chinensis* ecosystem functions. Furthermore, N addition may enhance the drought resistance of the *L. chinensis* meadow ecosystem to further drought events by increasing carbon allocation to roots and therefore root–shoot ratios.

## Author Contributions

WS and YW designed the experiment. YW, BM, and SZ performed the field and laboratory work, and BS analyzed the data. BS and WS wrote the manuscript.

## Conflict of Interest Statement

The authors declare that the research was conducted in the absence of any commercial or financial relationships that could be construed as a potential conflict of interest.
