# Supplementary material for: Effects of Nitrogen Addition on the Drought Susceptibility of the Leymus chinensis Meadow Ecosystem Vary with Drought Duration
Source: Front Plant Sci. 2018 Feb 27;9:254. doi: 10.3389/fpls.2018.00254 (PMC5835344; doi:10.3389/fpls.2018.00254)
Supplement: Supplementary file 1 [file Data_Sheet_1.docx]

**Effects of nitrogen addition on the drought susceptibility of the *Leymus chinensis* meadow ecosystem vary with drought duration**

***Baoku Shi^1^, Yunbo Wang^1,2^, Bo Meng^1^, Shangzhi Zhong^1^ & Wei Sun^1,*^***

*^1^ Key Laboratory for Vegetation Ecology, Ministry of Education, Institute of Grassland Science, Northeast Normal University, Changchun 130024, China*

*^2^ Key Laboratory of Grassland Resources, Ministry of Education, College of Grassland, Resources and Environment, Inner Mongolia Agricultural University, Hohhot 010018, China*

* Corresponding author

Wei Sun

[sunwei@nenu.edu.cn](mailto:sunwei@nenu.edu.cn)

Key Laboratory for Vegetation Ecology, Ministry of Education

Institute of Grassland Science, Northeast Normal University

Changchun, Jilin Province, P. R. China 130024

Tel.: +86 431 85098187


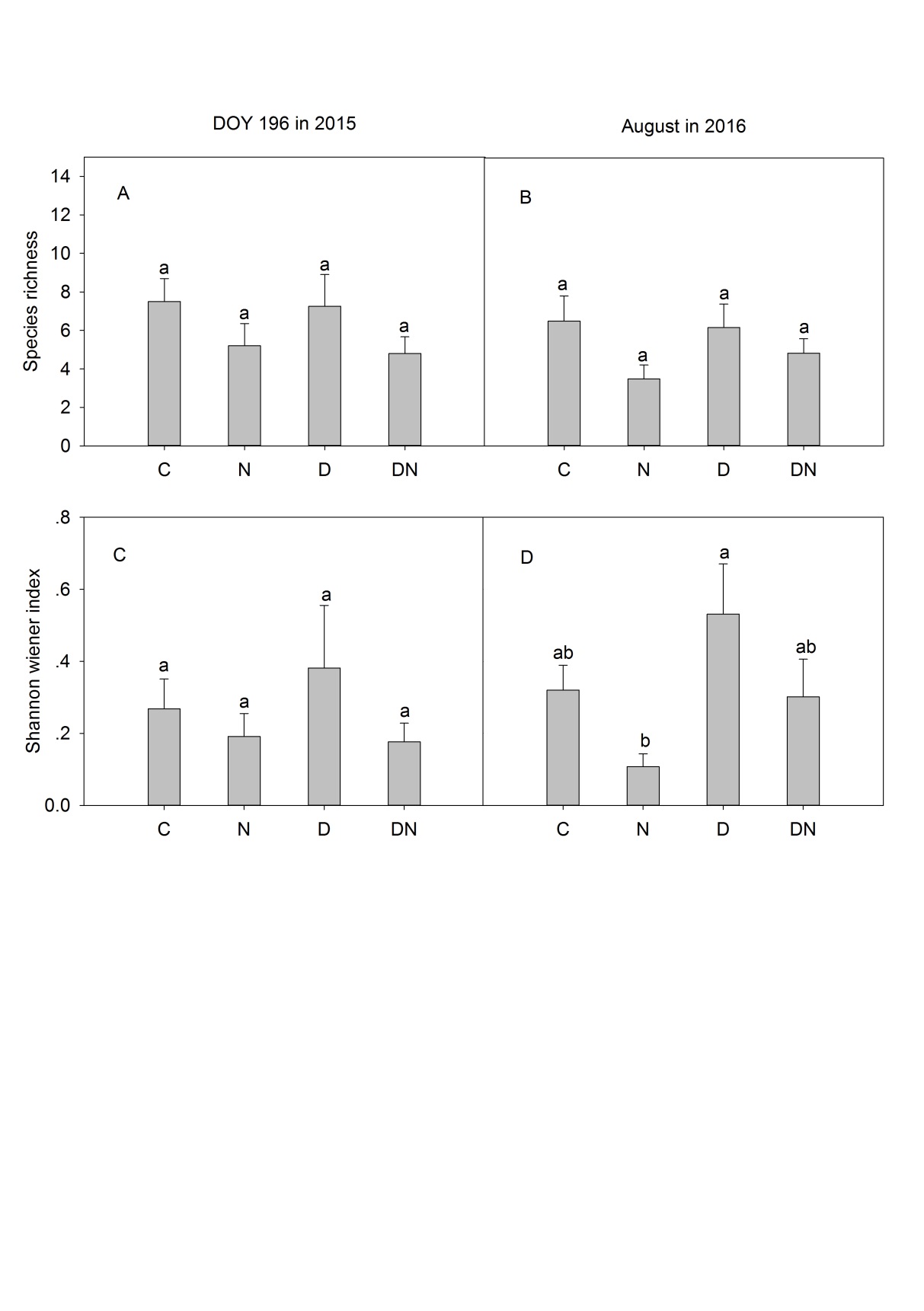


Figure S1 Responses of (A, B) species richness and (C, D) Shannon wiener index to four treatments (C: control, N: nitrogen addition, D: drought, DN: nitrogen addition plus drought) at the end of drought period (DOY 196 in 2015) and one year after the drought event (August in 2016). Different letters indicate significant differences between the treatments (*P* < 0.05). The error bars represent standard errors of means (n = 6).

Table S1 Mean relative growth rate of *Leymus chinensis* in different growth stages^*^.

| Growth stage | Jointing stage | Heading stage | Flowering stage | Milk-ripe stage | Soft dough stage | The end of soft dough stage |
| --- | --- | --- | --- | --- | --- | --- |
| Date (d/M) | 24/5 | 15/6 | 23/6 | 7/7 | 18/7 | 31/7 |
| Mean height (cm) | 14.1 | 22.4 | 28.0 | 33.1 | 36.0 | 38.1 |
| Mean relative growth rate (cm) | 0.38 | 0.70 | 0.35 | 0.26 | 0.16 | 0 |

^*^From Zhu (2004)

Table S2 Soil properties for the four treatments in 2014.

| Treatment | Soil total N Content (%) | Soil total P Content (%) | Soil pH | Soil electrical conductivity (µs cm^-1^) |
| --- | --- | --- | --- | --- |
| C | 0.13 (0.01)^B^ | 0.07 (0.01)^B^ | 9.16 (0.04)^A^ | 379 (23)^A^ |
| D | 0.11 (0.01)^C^ | 0.11 (0.01)^A^ | 9.05 (0.15)^A^ | 387 (20)^A^ |
| N | 0.16 (0.01)^A^ | 0.11 (0.01)^A^ | 8.99 (0.20)^A^ | 425 (37)^A^ |
| DN | 0.16 (0.01)^A^ | 0.11 (0.01)^A^ | 8.87 (0.23)^A^ | 432 (34)^A^ |

C: control, N: nitrogen addition, D: drought, DN: nitrogen addition plus drought; Different letters indicate significant differences between the treatments (*P* < 0.05).

Table S3 Results of the repeated-measures analysis of variance (ANOVA) on the effects of drought, nitrogen addition and measuring date and their interactions on leaf and ecosystem gas exchange parameters.

|  |  | *A* | |  | *g*_s_ | |  | *T*_r_ | |  | GEP | |  | ER | |  | NEE | |  | ET | |  | WUE | |
| --- | --- | --- | --- | --- | --- | --- | --- | --- | --- | --- | --- | --- | --- | --- | --- | --- | --- | --- | --- | --- | --- | --- | --- | --- |
| Treatment | *df* | *F* | *P* |  | *F* | *P* |  | *F* | *P* |  | *F* | *P* |  | *F* | *P* |  | *F* | *P* |  | *F* | *P* |  | *F* | *P* |
| D | 1 | 86.8 | **<0.01** |  | 10.7 | **<0.01** |  | 11.0 | **<0.01** |  | 33.5 | **<0.01** |  | 19.4 | **<0.01** |  | 36.3 | **<0.01** |  | 29.8 | **<0.01** |  | 0.2 | 0.70 |
| N | 1 | 3.04 | 0.10 |  | 2.6 | 0.12 |  | 5.1 | **0.04** |  | 36.6 | **<0.01** |  | 14.4 | **<0.01** |  | 47.7 | **<0.01** |  | 6.0 | **0.03** |  | 14.7 | **<0.01** |
| T | 1 | 32.7 | **<0.01** |  | 24.5 | **<0.01** |  | 35.6 | **<0.01** |  | 30.4 | **<0.01** |  | 14.8 | **<0.01** |  | 29.8 | **<0.01** |  | 40.9 | **<0.01** |  | 0.8 | 0.40 |
| D×N | 1 | 0.1 | 0.93 |  | 0.1 | 0.74 |  | 0.3 | 0.60 |  | 3.6 | 0.07 |  | 1.6 | 0.23 |  | 4.5 | **0.04** |  | 0.1 | 1.00 |  | 0.5 | 0.47 |
| D×T | 1 | 0.2 | 0.67 |  | 0.1 | 0.86 |  | 0.1 | 0.77 |  | 2.0 | 0.17 |  | 2.0 | 0.17 |  | 1.3 | 0.27 |  | 0.2 | 0.69 |  | 0.3 | 0.61 |
| N×T | 1 | 4.7 | 0.04 |  | 0.1 | 0.78 |  | 0.1 | 0.74 |  | 11.8 | **<0.01** |  | 5.5 | **0.03** |  | 11.8 | **<0.01** |  | 6.1 | **0.03** |  | 0.1 | 0.71 |
| D×N×T | 1 | 0.1 | 0.89 |  | 0.7 | 0.43 |  | 0.7 | 0.42 |  | 1.0 | 0.32 |  | 0.1 | 0.74 |  | 2.8 | 0.11 |  | 1.3 | 0.26 |  | 0.3 | 0.59 |

Bold values denote significant differences at *P* < 0.05. D: drought, N: nitrogen addition, T: time, *A*: leaf net CO_2_ assimilation rate, *g*_s_: stomatal conductance, *T*_r_: transpiration rate, GEP: gross ecosystem productivity, ER: ecosystem respiration, NEE: net ecosystem CO_2_ exchange, ET: evapotranspiration, WUE: water-use efficiency.

**REFERENCES**

Zhu, T. C. (2004). *Yang-cao Biological Ecology* (in Chinese). Changchun: Jilin Science and Technology Press.
